# Anti-inflammatory and morphologic effects of pitavastatin on carotid arteries and thoracic aorta evaluated by integrated backscatter trans-esophageal ultrasound and PET/CT: a prospective randomized comparative study with pravastatin (EPICENTRE study)

**DOI:** 10.1186/s12947-015-0012-9

**Published:** 2015-04-02

**Authors:** Takatomo Watanabe, Masanori Kawasaki, Ryuhei Tanaka, Koji Ono, Nobuo Kako, Maki Saeki, Noriyuki Onishi, Maki Nagaya, Noriaki Sato, Hirotaka Miwa, Masazumi Arai, Toshiyuki Noda, Sachiro Watanabe, Shinya Minatoguchi

**Affiliations:** Department of Cardiology, Gifu University Graduate School of Medicine, 1-1 Yanagido, Gifu, 501-1194 Japan; Department of Cardiology, Gifu Prefectural General Medical Center, Gifu, Japan; Aichi Health Screening Association, Nagoya, Japan

**Keywords:** Statin, Carotid artery, Aorta, Positron emission tomography, Ultrasound

## Abstract

**Background:**

We sought to evaluate the effects of a strong lipophilic statin (pitavastatin) on plaque components and morphology assessed by transesophageal echocardiography (TEE) and transthoracic echocardiography (TTE), as well as plaque inflammation assessed by 18 F-fluorodeoxyglucose (FDG) PET/CT in the thoracic aorta and the carotid artery. Furthermore, we compared the effects of pitavastatin with those of mild hydrophilic statin (pravastatin).

**Methods:**

We examined atherosclerotic plaques in the thoracic aorta by TEE and those in the carotid artery by integrated backscatter (IBS)-TTE and PET/CT. We identified the target plaque, where there was macrophage infiltration and inflammation, by strong FDG uptake in the thoracic aorta and carotid arteries and measured maximum standard uptake values (max SUV) by PET/CT. We measured the intima-media thickness (IMT) and the corrected IBS (cIBS) values in the intima-media complex by TEE and TTE at the same site of FDG accumulation by PET/CT.

**Results:**

Patients were randomly divided into two treatment groups: a pitavastatin group (PI group: n =10, 68.4 ± 5.1 years) and a pravastatin group (PR group: n =10, 63.9 ± 11.2 years). The same examinations were performed after six months at the same site in each patient. We used calculated target-to-background ratio (TBR) to measure max SUV of plaques and evaluated percent change of TBR. There was no significant difference in low density lipoprotein-cholesterol, TBR, IMT and cIBS values in plaques at baseline between the PI and PR groups. After treatment, there was greater improvement in TBR, cIBS values and IMT in the PI group than the PR group.

**Conclusions:**

The pravastatin treatment was less effective on plaque inflammation than pitavastatin treatment. This trend was the same in the carotid arteries and the thoracic aorta. Pitavastatin not only improved the atherosis as measured by IMT and cIBS values but also attenuated inflammation of plaques as measured by max SUV at the same site. The present study indicated that pitavastatin has stronger effects on the regression and stabilization of plaques in the thoracic aorta and carotid arteries compared with pravastatin.

## Introduction

HMG-Co-A reductase inhibitor drugs (statins) reduce the mortality of myocardial infarction and stroke, and prevent the progression of atherosclerosis [1,2]. Moreover, it is well known that statins have pleiotropic effects and there are many reports on these effects [3,4]. We previously reported that ultrasound integrated backscatter (IBS) can discriminate tissue characteristics of carotid plaques, and IBS values of carotid media were correlated with arterial stiffness and the degree of fragmentation of elastic fiber in carotid media [5,6]. Using the IBS technique, we also reported that pitavastatin improved atherosclerotic changes in the media of the thoracic aorta [7]. Although there have been several studies that examine the effects of statins on atherosclerotic plaque inflammation or plaque volume, there are few studies that examined both morphologic and anti-inflammatory effects of statins on atherosclerotic lesions.

18 F-fluorodeoxyglucose (FDG) is able to accumulate in plaque macrophages [8]. FDG-positron emission tomography (PET) imaging of atherosclerotic plaques can be clinically used to assess the severity of inflammation in plaques and is highly reproducible [9-11]. Previous studies demonstrated that simvastatin attenuated 18 F-FDG uptake in carotid plaques and decreased the standardized uptake values (SUVs), and atorvastatin also reduced 18 F-FDG uptakes in atherosclerotic plaques [12,13].

Statins are pharmacologically divided into hydrophilic and lipophilic agents. Furthermore, it has been suggested that the strength of pleiotropic effects may vary depending on a statin’s characteristics. However, there have been few studies that have directly compared hydrophilic and lipophilic statins. The purpose of the present study was (1) to evaluate the effects of a strong lipophilic statin (pitavastatin) on plaque components and morphology assessed by IBS transesophageal echocardiography (TEE), as well as plaque inflammation assessed by 18 F-FDG positron emission tomography/computed tomography (PET/CT) in the carotid artery and thoracic aorta, and (2) to compare the effects of pitavastatin with those of a mild hydrophilic statin (pravastatin).

## Methods

### Study population

Patients who satisfied the following criteria were enrolled in the present study: 1) patients with hyperlipidemia [total cholesterol ≥220 mg/dl and/or low density lipoprotein (LDL) cholesterol ≥140 mg/dl, as defined by the Japan Atherosclerosis Society] or who needed lipid lowering therapy with statin based on the Japanese guidelines for the prevention of atherosclerotic disease (1–2 coronary risk factors: an LDL cholesterol ≤140 mg/dl; 3–4 coronary risk factors: an LDL cholesterol ≤120 mg/dl; or a history of ischemic heart disease despite an LDL cholesterol ≤100 mg/dl) and had no treatment with statins in the six months before enrollment, 2) patients with a high risk of atherosclerosis such as a severe plaque observed in the aorta or a carotid artery by past examination using CT or ultrasound, an aortic calcification observed through a chest x-ray, or metabolic syndrome, and 3) patients who required TEE due to atrial fibrillation or valvular heart disease. Patients were excluded if they had active infectious disease, poorly controlled diabetes, renal dysfunction or cancer found previously.

### Study protocol

This was a prospective, randomized and comparative study called EPICENTRE study (Anti-inflammatory and Morphologic Effects of Pitavastatin on Carotid Arteries and Aorta Evaluated by Integrated Backscatter Trans-esophageal Ultrasound and PET/CT).

Patients were randomly divided into two groups: a pitavastatin treatment group (PI group: 2 mg/day), and a pravastatin treatment group (PR group: 10 mg/day). The dose of each statin (2 mg/day of pitavastatin and 10 mg/day of pravastatin) is intermediate dose in Japan. They each received treatment for six months, and the same examinations were performed after treatment at the same site in each patient. Blood biochemical examination was also conducted to assess lipid profile, glucose metabolism and the side effects of each medicine before and after treatment. This study protocol was approved by the ethics committee of our institution. Written informed consent was obtained from all enrolled patients.

### PET/CT image acquisition and analysis

PET/CT images were obtained using methods as described in a previous study [5,7-10]. Patients were injected with 3.7 MBq /kg (0.1 mCi /kg) of 18 F-FDG intravenously. The injection was given after fasting for at least 8 hours. After one hour, two-dimensional whole-body PET/CT imaging was performed using a PET/CT scanner (Discovery ST Elite Performance, GE Medical Systems, Milwaukee, WI). This system can obtain PET and CT images (4.25-mm slice thickness) simultaneously. CT scan was performed for attenuation correction and PET images were reconstructed using iteration algorithm (128 × 128 pixel matrix). Images were obtained over 24 minutes. 18 F-FDG uptake was measured using a workstation (XTREX VIEW, J-MAC system, Japan).

At baseline, we manually found two regions that had a strong intensity of 18 F-FDG uptake in the thoracic aorta and carotid arteries in each patient and we defined them as target plaques. A region of interest (ROI) was placed on the target plaque as a spherical region 10 mm in diameter. We measured maximum SUV (max SUV) of the ROI and considered it as the max SUV of the target plaque. A target-to-background ratio (TBR) has recently been used as a method of normalization of the SUVs. It represents 18 F-FDG uptake by macrophages in inflamed vascular cells [4]. The TBR was calculated as the max SUV of target plaques divided by the mean SUV of blood which was measured in the right atrium. We were able to find target plaque easily after treatment using the distance from significant landmarks on PET/CT images (e.g., calcification of arterial wall, corpus vertebrae and bifurcations of an artery). The analyses of PET/CT were conducted by one radiologist and one cardiologist, who were blinded to the patients’ treatment assignment. The average of two measurements was used for the analysis. After six months later, we selected the same region as that selected at baseline by refereeing CT images.

### Transesophageal echocardiography and conventional ultrasonography

TEE and conventional ultrasonography of carotid arteries were performed to measure intima-media thickness (IMT) and the corrected IBS (cIBS) values in the intima-media complex of the target plaques within two weeks after PET/CT. The IBS values in the intima-media complex were corrected by subtracting the IBS values in the tunica externa as follows [14]:

$$ \mathrm{cIBS}=\mathrm{I}\mathrm{B}\mathrm{S}\ \mathrm{values}\ \mathrm{in}\ \mathrm{the}\ \mathrm{aortic}\ \mathrm{wall} - \mathrm{I}\mathrm{B}\mathrm{S}\ \mathrm{values}\ \mathrm{in}\ \mathrm{the}\ \mathrm{tunica}\ \mathrm{externa}. $$

TEE was performed with an ultrasonic imaging system (SONOS 5500, Philips Medical Systems, Andover, MA, USA) and a 4–7 MHz multi-plane transducer with a 7.4 mm diameter “pediatric probe” (T6207, Philips Medical Systems, Andover, MA, USA) in the echocardiography laboratory by an operator who was blinded to the patients’ treatment assignment. The oropharynx was anesthetized with lidocaine before esophageal intubation. After the cardiac examination, the transducer was rotated in a posterior direction to obtain aortic images. The cIBS values in the intima and media of a target plaque and the intima-media thickness (IMT) at the same site were measured. The distance between the target plaque and the bifurcation of the left subclavian artery was measured by counting slices of CT, and target plaques were anatomically detected by TEE. If there was a characteristic calcification near the target plaque, it was also recorded for use as a reference point. The plaque was detected by this method with high reproducibility. Conventional ultrasound images and cIBS values of the carotid arteries were easily acquired at the same time using the same ultrasonic imaging system and a 3–11 MHz linear-array transducer.

We calculated average IMT and cIBS values of 5 slices spaced at-1 mm intervals around the target plaques. We detected two target plaques from thoracic aorta and two target plaques from right and left carotid arteries in each patient.

### Measurement of c-IBS

IBS analysis was performed with a software package “Acoustic Densitometry” with the SONOS 5500. In this system, cIBS values are calculated as the average power of the ultrasonic backscattered signal from the region-of-interest (ROI) and represent the tissue characteristics. When measuring IBS values of plaques, ROIs were set on just the inner side of the tunica externa. The ROIs (21 × 21 pixels, 1.1 × 1.1 mm) placed at this site covered only intimal plaques.

The method of measuring IBS values was the same we previously reported [13]. We calculated averaged IMT and cIBS of 5 slices at 1-mm intervals around the target plaque.

### Statistical analysis

Data are expressed as the mean ± one standard deviation or the number of patients (percentage). The normality of distribution was tested using the Kolmogorov-Smirnov test. The significance of the differences between groups that were normally distributed and had similar variances was tested by an unpaired Student’s *t* test. Otherwise, a Mann–Whitney *U* test was used to compare the difference between groups. Categorical data were summarized as percentages and compared using a Chi-square test or Fisher exact test. The relationships between the change of lipid profile and the change of TBR were tested for significance by linear regression analysis. All statistical analyses were performed using Stat View version 5.0 (SAS Institution Inc., Cary, NC, USA). A p-value < 0.05 was considered significant.

## Results

### Patients baseline characteristics and biochemical parameters

We enrolled 24 patients in the present study. One patient dropped out voluntarily. Three patients were excluded because of worsening diabetes, active infectious disease in the follow-up period and an incidental detection of pancreatic cancer on the first PET/CT. Finally 20 patients were examined (PI group: n =10, 68 ± 5 years; PR group: n =10, 64 ± 11 years). Baseline clinical characteristics of the patients are shown in Table 1. There were no significant differences in age, height, body weight, underlying disease and smoking rates between the PI and PR groups at baseline.

**Table 1:** **Patients’ characteristics at baseline**

	**Pitavastatin (n = 10)**	**Pravastatin (n = 10)**	**p value**
Age, year	68 ± 5	64 ± 11	0.26
Man, n (%)	7 (70)	8 (80)	>0.99
Height, cm	161.3 ± 6.1	162.5 ± 11.3	0.78
Body weight, kg	65.0 ± 9.1	63.5 ± 10.6	0.73
Main history, n (%)			
Hypertension	5 (50)	5 (50)	>0.99
Diabetes mellitus	4 (40)	2 (20)	0.36
Smoking, n (%)	6 (60)	5 (50)	0.67
Valvular heart disease	3 (30)	2 (20)	0.63
Medications, n (%)			
ARBs or ACEIs	2 (20)	5 (50)	0.18
Beta-blockers	3 (30)	3 (30)	>0.99
Ca channel blockers	4 (40)	3 (30)	0.67

ARBs: angiotensin II receptor blockers, ACEIs: angiotensin-converting enzyme inhibitors, Ca: calcium.

Baseline biochemical measurements in both groups are shown in Table 2. There were no significant differences in lipid level, liver-associated enzymes, creatinine phosphokinase and high-sensitive C-reactive protein (hs-CRP) between the two groups. There were also no differences between the two groups in other baseline parameters, such as cIBS values, IMT and TBR, also showed no significant difference between the two groups (Table 1). No patient experienced any adverse reactions such as elevation of liver-associated enzymes (3 times upper limit of normal) or myositis. There were no differences in baseline underlying diseases and were no serious cardiovascular events including myocardial infarction, unstable angina, or death in either group during the follow-up period.

**Table 2:** **Laboratory parameters**

	**Pitavastatin**		**Pravastatin**	
	**Baseline**	**After 6 months**	**Baseline**	**After 6 months**
Total cholesterol, mg/dl	202 ± 67	154 ± 22*^†^	225 ± 21	185 ± 16*
LDL cholesterol, mg/dl	150 ± 21	80 ± 16*†	142 ± 24	103 ± 18*
HDL cholesterol, mg/dl	52 ± 12	52 ± 11	54 ± 15	55 ± 13
Triglyceride, mg/dl	134 ± 35	107 ± 52	167 ± 63	135 ± 71
AST, IU/l	28 ± 13	26 ± 5	34 ± 18	32 ± 13
ALT, IU/l	27 ± 17	25 ± 9	29 ± 15	30 ± 12
γ-GTP, IU/l	34 ± 19	38 ± 28	79 ± 89	76 ± 84
CK, IU/l	77 ± 31	85 ± 26	95 ± 53	108 ± 63
High-sensitive CRP, μg/l	2,883 ± 4,176	3,581 ± 8,445	1,747 ± 2,239	3,928 ± 5,025
Hemoglobin A1c, %	5.8 ± 0.8	5.9 ± 0.7	5.4 ± 0.5	5.5 ± 0.4
Insulin, μU/ml	7.0 ± 4.7	6.7 ± 3.5	5.4 ± 2.9	4.7 ± 2.6

LDL: low density lipoprotein, HDL: high density lipoprotein, AST: aspartate aminotransferase, ALT: alanine aminotransferase, CRP: C-reactive protein, CK: creatinine phosphokinase, *p < 0.05 vs baseline, ^†^p < 0.05 vs pravastatin.

### Changes after 6 months of statin therapy

After 6 months, total cholesterol and LDL-cholesterol in the PR group were decreased from 225 ± 21 to 185 ± 16 mg/dl and from 142 ± 24 to 103 ± 18 mg/dl, respectively. The total cholesterol and LDL-cholesterol in the PI group significantly decreased from 202 ± 67 to 154 ± 22 mg/dl and from 150 ± 21 to 80 ± 16 mg/dl, respectively. There was no significant difference in hs-CRP at baseline or six months, or in the change from baseline to six months between the two groups, because there were relatively large variances of the data. There were no significant alterations in glucose metabolism between baseline and after 6 months in the two groups (Table 2).

**Figure 1:**
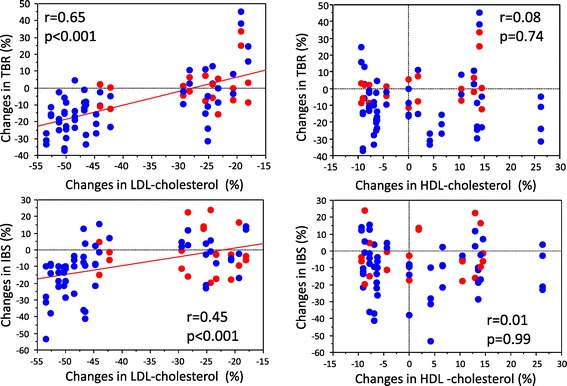
**Correlation between the changes in each parameter from baseline to 6 months and the changes in low density lipoprotein cholesterol and high density lipoprotein cholesterol.** Red dots indicate pravastatin group and blue dots indicate pitavastatin group.

**Figure 2:**
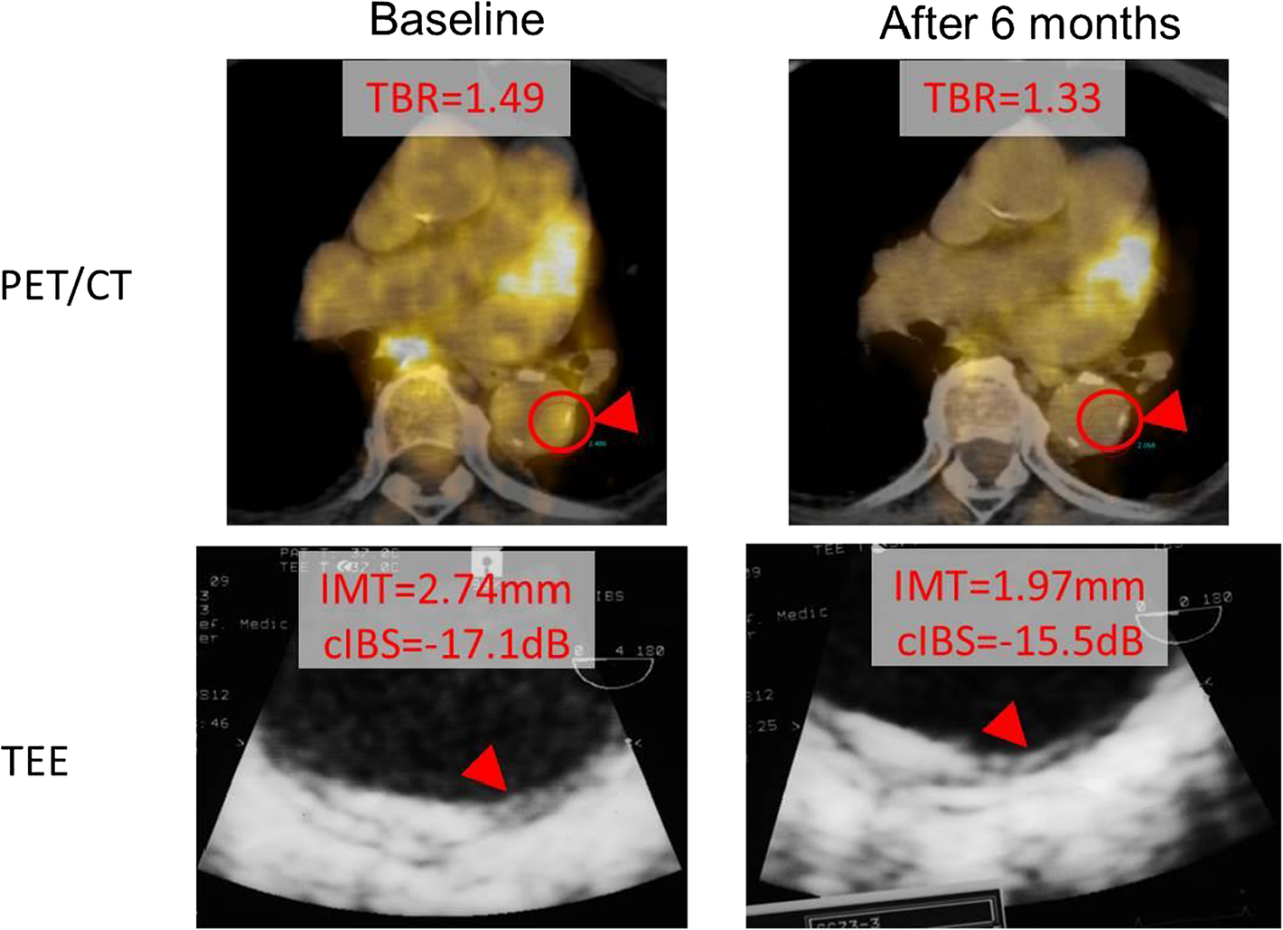
**PET: positron emission tomography, TEE: trans-esophageal echocardiography, TBR: target-to-background ratio, IMT: intima-media thickness, cIBS: corrected integrated backscatter.**

**Figure 3:**
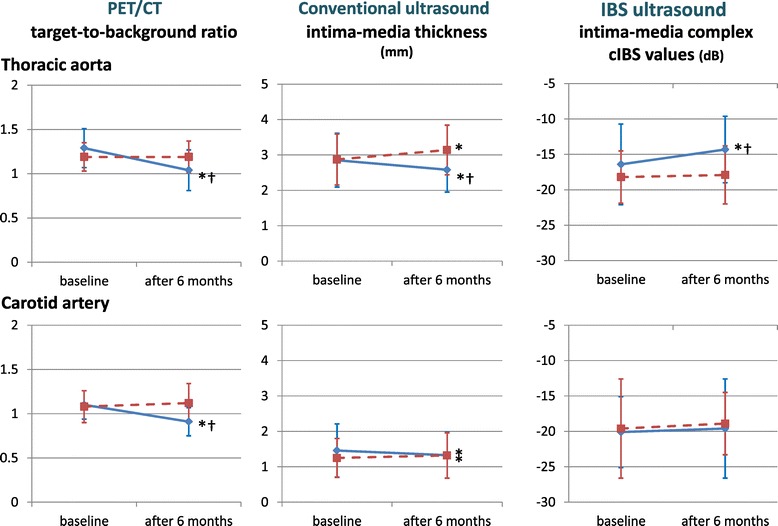
PET/CT and ultrasound parameters, TBR: target-to-background ratio, IMT: intima-media thickness, cIBS: corrected integrated backscatter, *p < 0.05 vs baseline, ^**†**^ **p < 0.05 vs pravastatin.**

The TBRs of the plaques were significantly decreased from 1.29 ± 0.22 to 1.04 ± 0.23 in the PI group, whereas TBRs did not decrease in the PR group (from 1.19 ± 0.16 to 1.19 ± 0.18). The cIBS values in the intima-media complex of the plaques in the PI group increased significantly more than those in the PR group (−16.4 ± 5.7 dB to −14.3 ± 4.7 dB and −18.2 ± 3.7 dB to −17.9 ± 4.1 dB, respectively). The IMT of the plaques in the PI group was significantly reduced, but the IMT in the PR group increased (2.85 ± 0.76 mm to 2.58 ± 0.63 mm and 2.87 ± 0.72 mm to 3.14 ± 0.7 mm, respectively). There were significant improvements of TBR, cIBS and IMT in the PI group but there was less effect on these parameters in the PR group (Figures 1, 2, 3). This trend was the same also in the carotid arteries and the thoracic aorta (Figure 3). The changes of TBR and cIBS values between baseline and 6 months were significantly correlated with the change of LDL cholesterol (r = 0.65 and r = 0.45, respectively), but not the change of HDL cholesterol (Figure 1).

## Discussion

The present study compared the effects of a strong lipophilic statin (pitavastatin) on plaque components and morphology as well as plaque inflammation. The present study also demonstrated that statin decreased plaque volume measured by intima-media thickness and contributed to plaque stabilization measured by cIBS values. Furthermore, pitavastatin stabilized plaque inflammation (evaluated by TBR) more than pravastatin in both the carotid artery and the thoracic aorta.

In addition, we demonstrated that the change of cIBS values and TBR were correlated with the change of LDL cholesterol, but not with the change of HDL cholesterol. Tahara et al. suggested that attenuation of vascular inflammation may be associated with an increase in HDL cholesterol rather than with LDL cholesterol in a study using simvastatin [9]. On the other hand, Ishii H et al. reported that decreased TBR was correlated with a reduction of LDL cholesterol [10]. In the present study, we found a correlation between a change of cIBS values and TBR and a change of LDL cholesterol rather than that of HDL cholesterol. In addition, a strong lipophilic statin decreased LDL cholesterol and changed TBR and cIBS values more than a mild hydrophilic statin. Other investigators reported that there was a significant correlation between LDL cholesterol lowering effects and anti-inflammatory effects [15]. The findings in the present study reinforced the previous findings.

It is known that high plasma hs-CRP concentrations are associated with high cardiovascular risk. In addition, hs-CRP was decreased in diabetic patients treated with pitavastatin regardless of a change in serum lipid profile [16]. However, in the present study, we could not establish a relationship in hs-CRP because there were relatively large variances of the hs-CRP. It was speculated that the reasons were the nonspecificity of hs-CRP and small sample size in the present study. Actually, arthritis and enteritis were detected by PET/CT in some enrolled patients in the present study.

It is thought that lipophilic statin diffuses more directly to vascular endothelial cells and has a stronger pleiotropic effect than hydrophilic statin [3]. Treatment for six months in the present study with a mild hydrophilic statin improved neither TBR nor IBS, and IMT significantly increased from baseline. There was a similar report that pravastatin resulted in less regression of the carotid artery IMT than rosuvastatin [17]. The present study suggests that it is better to use a strong lipophilic statin rather than a mild hydrophilic statin to prevent or cause regression of atherosclerosis in the clinical setting.

In the present study, we obtained PET images using relatively short duration (one hour) after the injection of FDG. There were several studies that employed various durations between the injection of FDG and PET imaging for the evaluation of atherosclerotic lesions (1–3 hours) [9,12,18]. Studies that employed long duration between the injection and imaging aimed to include the change of proper uptake by macrophages as well as better washout of radioactivity from the blood pool. Contrary, we aimed to include the change of the high residual activity in neighboring pixels due to the short injection-to-imaging time.

### Study limitations

There are several limitations of this study. First, the number of patients in our analysis was small. Our results and conclusions require additional validation in a larger population. Second, this study was based on the assumption that cIBS values of the thoracic aorta reflect the same type of histological changes indicated by the cIBS values of carotid artery that have already been validated. Validation between the cIBS values and tissue components in the thoracic aorta are required. Third, we did not use Agatston score for the evaluation of atherosclerosis because the CT protocol was used only for PET attenuation correction. However, process of progression of calcification is gradual and not suitable for the relatively acute change by the effects of statins. Fourth, a small size of the ROI chosen for PET quantification in the present study may hinder accurate reproducibility of measurements. Relatively large ROIs should be used to improve the reproducibility of measurements.

## Conclusions

Pitavastatin not only improved atherosis measured by IMT and the sclerosis measured by cIBS values, but also attenuated inflammation of plaques at the same site as measured by max SUV. The present study indicated that pitavastatin had a stronger effect on regression and stabilization of plaques in the thoracic aorta compared with a mild hydrophilic statin.

## Abbreviations

TEE: Transesophageal echocardiography
TTE: Transthoracic echocardiography
FDG: 18 F-fluorodeoxyglucose
PET/CT: Positron emission tomography/computed tomography
IBS: Integrated backscatter
LDL: Low density lipoprotein, HDL, High density lipoprotein
hs-CRP: High-sensitive C-reactive protein
SUV: Standard uptake values
IMT: Intima-media thickness
cIBS: Corrected IBS
TBR: Target-to-background ratio
